# Prevalence of Portable Point of Care Tests Used on Medical Service Trips in Latin America and the Caribbean

**DOI:** 10.29024/aogh.2385

**Published:** 2018-11-05

**Authors:** Christopher Dainton, Nikki Shah, Charlene H. Chu

**Affiliations:** 1 McMaster University, CA; 2 Grand River Hospital, Kitchener, CA; 3 Medical Service Trip.com, CA; 4 McMaster University School of Medicine, CA; 5 Toronto Rehabilitation Institute-University Health Network, CA

## Abstract

**Background:**

Short-term, primary care medical service trips (MSTs) frequently use inexpensive, portable point of care (POC) tests to guide diagnosis and treatment of patients in low-resource settings. However, the degree to which different POC tests are carried by organizations serving remote communities is currently unknown.

**Objective:**

The aim of this study was to determine the prevalence of various POC tests used by MST-sending organizations operating in Latin America.

**Methods:**

We surveyed 166 organizations operating mobile MSTs in Latin America and the Caribbean on the types of POC tests carried on their brigades.

**Findings:**

Forty-eight organizations responded (response rate: 28.9%). The most commonly carried tests were glucometers (40/48; 83.3%), urine dipsticks (31/48; 77.1%), and urine pregnancy tests (32/48; 66.7%). Fewer groups carried hemoglobinometers (16/48; 33.3%), malaria diagnostic tests (18/48; 37.5%), tests for sexually transmitted infection (8/48; 16.7%), or portable ultrasound (19/48; 40.0%).

**Conclusions:**

These tests may be useful for field diagnosis, but clinicians should understand the performance limitations of each test compared to its gold standard. When combined with knowledge of local epidemiology, these exploratory results will be useful in resource planning, guidelines development for MSTs, and in establishing minimum recommendations for diagnostic resources that should be available on MSTs.

## Introduction

The prevalence of primary care medical service trips (MSTs) operating in low- and middle-income countries (LMICs) abroad has increased dramatically in recent years [1,2], and Latin America is a common destination. The delivery of care on short-term MSTs commonly involves establishing mobile clinics in various non-medical settings ranging from churches to schoolhouses or community buildings. The logistics and cost of transporting diagnostic supplies and equipment between remote clinic sites mean that organizations operating such trips are generally limited to use of inexpensive, portable point of care (POC) tests that provide immediate results to inform treatment decisions [3,4]. Furthermore, there are few clinical guidelines that provide a foundation for evidence-based practice on such trips [5].

POC tests play an important role in patient management, particularly where formal laboratory tests (e.g. microscopic urinalysis or blood cultures) or clinical follow-up may be difficult to obtain. Common examples include urine dipstick testing for the diagnosis of urinary tract infection (UTI), hyperglycemia, or proteinuria; glucometer testing for diabetes; qualitative urine pregnancy tests; and use of a hemoglobinometer for obtaining a hemoglobin. These tests may replace gold standard laboratory methods while practicing in remote settings.

The purchase of supplies for mobile clinics is a significant cost consideration for MST-sending organizations, especially given the increasing number of commercially available POC devices, ranging from ultrasound, to testing for infectious agents such as streptococcus, chlamydia, HIV, malaria, and others [4,6]. In the context of MSTs and the low-resource environments that they serve, there are opportunity costs in choosing one POC test over another. In a resource-limited setting, organizations must aim to carry only those supplies that offer the greatest utility for their clinicians and, ultimately, benefit for patients. It is currently unknown which types of tests are commonly carried by volunteers serving remote communities on MSTs, making future investigations regarding their relative efficiency and utility for diagnosis in the field difficult. It is also unclear whether level of engagement, motivation, or location of service influence the number or type of POC tests that these groups carry.

The purpose of this study is to describe the prevalence of common POC tests carried by primary care mobile MSTs serving Latin America and the Caribbean (LAC), and possible associations between type of tests carried and the characteristics of the MSTs themselves. This information is important to understand the diagnostic capacity and capabilities of MSTs in remote settings, and provide new and veteran MST-sending organizations with an impression of the minimum resources that would be expected on a mobile MST.

## Methods

This study used a descriptive exploratory approach to collect cross-sectional email survey data from MST-sending organizations from February 10, 2016 to May 30, 2016.

### Database construction

MST-sending organizations currently operating MSTs in LAC were identified in three ways. First, databases of MST-sending organizations were used (www.missionfinder.org, www.medicalmissions.org, www.mmex.org, www.globalhealth.arizona.edu, www.internationalhealthvolunteers.org) to identify relevant organizations. Second, a systematic Google search [7] was conducted using the terms: medical missions, short-term missions, and medical mission organizations, combined with country of service. Based on Lasker [2], the Google search was extended to include additional combinations of terms, including: “international health volunteering”, “Christian health volunteering”, “religious health volunteering”, “corporate global health volunteering”, “international health fellowships”, “international health educational opportunities”, “global health director”, “international service learning”, “global health elective”, “medical school international internships”, “intercultural learning”, “global health volunteer projects university”, and “international volunteer organizations”. Third, organizations were located through Twitter, using the hashtags “medical mission” and “global health”. These searches were performed several times between April 17, 2014 and July 20, 2015 to include as many organizations as possible, due to the diversity of web presence and fluidity of the MST landscape.

Organizations were included in the database if their websites indicated that they: facilitated the provision of direct patient care by North American clinicians (physician, physician assistant, osteopath, or nurse practitioner) in LAC and had operated at least one MST in the previous 12 months. Organizations were specifically excluded if they: exclusively performed specialty trips, surgical trips, trips that did not involve a mobile clinic component, or trips of more than one-month duration. Organizations with multiple chapters (i.e. university MST organizations) were treated as one unified parent organization.

### Data collection and analysis

We extracted the following information from each organizational website: the location of their headquarters in North America, the location(s) served in LAC, the frequency of MSTs to LAC, setting of their mobile clinics, the number and type of providers, and whether the organization was faith-based.

All eligible organizations were contacted via email by a research assistant to request a list of POC diagnostic tests that they routinely used in their mobile clinics. They were specifically asked if they carried urine dipsticks, qualitative urine pregnancy tests, hemoglobinometers, glucometers, portable ultrasound, malaria diagnostic testing, or testing for sexually transmitted infections (i.e. chlamydia) and were given an opportunity to add any additional POC tests to which they had access. The Dillman approach was used, which relies on personalized, repeated contact with the organizations (e.g. “Dear Justin”) to maximize survey response rates [8]. The information requests included the purpose of the study, its potential benefit to the literature around MSTs, and contact information for the authors. Two reminder emails were sent to partial and non-respondents approximately two and four weeks after the initial survey was sent.

Descriptive data for each organization and survey responses regarding POC test prevalence were entered in an Excel file and analysed. Bivariate statistical analysis (2-sided Fisher exact test for equality between groups) was performed to compare the characteristics of the MST-sending organizations to the types of POC tests carried and to determine whether there were differences between faith-based and secular groups, or between groups who served Latin America frequently versus those who visited rarely. Analysis included only the available data; no imputation was conducted to replace missing data. Significance was defined as a p-value of ≤0.05.

## Results

**Figure 1:**
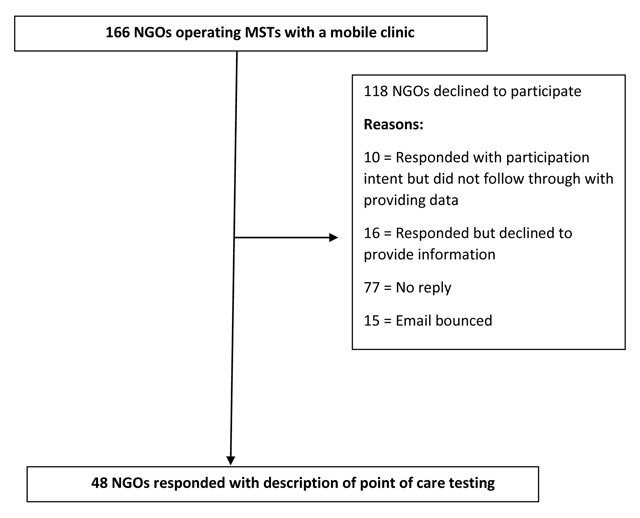
Flow chart of organizations operating mobile MSTs who were asked to describe their point of care testing resources.

The search resulted in 166 MST-sending organizations operating short-term primary care mobile clinics in LAC. All organizations were contacted by email for information on their POC testing resources, and 48 organizations responded (Figure 1; response rate = 28.9%). Table 1 compares the characteristics of the respondents to the overall pool of organizations solicited.

**Table 1:** Characteristics of survey respondents versus those solicited in a database of 166 organizations sending mobile MSTs to Latin America and the Caribbean (percentages do not add to 100% in cases where organizations served more than one region).

	NGOs contacted (n = 166) n, (%)	NGOs responding (n = 48) n, (%)

**Locations served**		
*Central America*	92 (55.4%)	29/48 (60.4%)
*Caribbean*	64 (38.6%)	16/48 (33.3%)
*South America*	40 (24.1%)	13/48 (27.1%)
*Unknown*	2 (1.2%)	1/48 (2.1%)

**Trip Frequency**		
>*3 trips/year*	64 (38.6%)	20/48 (41.7%)
<*3 trips/year*	556 (33.1%)	21/48 (43.7%)
*Unknown*	17 (10.2%)	7/48 (14.6%)

**Religious affiliation**		
*Faith based*	102 (61.5%)	29/48 (60.4%)
*Secular*	57 (34.3%)	17/48 (35.4%)
*Unknown*	7 (4.2%)	2/48 (4.2%)

**Setting**		
*Rural*	127 (76.5%)	44/48 (91.7%)
*Unknown*	36 (21.7%)	3/48 (6.3%)

### POC test prevalence

**Figure 2:**
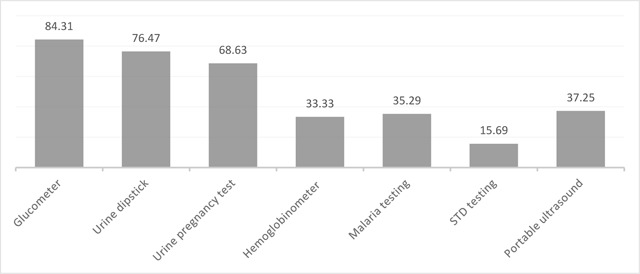
Percentage of organizations operating mobile MSTs in Latin America and the Caribbean carrying selected point of care tests (N = 48).

Figure 2 depicts the prevalence of each type of POC test. The most commonly carried tests were glucometers (n = 40; 83.3%), urine dipsticks (n = 31; 77.1%), and qualitative urine pregnancy tests (n = 32; 66.7%). Fewer organizations carried hemoglobinometers (n = 16; 33.3%), malaria diagnostic tests (n = 18; 37.5%), or portable ultrasound (n = 19; 40.0%). The least commonly carried of the POC tests specifically listed in the survey were those for diagnosis of chlamydia (n = 8; 16.7%). Other tests mentioned by the organizations but not specifically included in our original list included electrocardiography (ECG; n = 6; 12.5%); iSTAT/blood count (n = 5; 10.4%); human immunodeficiency virus (HIV) testing (n = 4; 8.3%); microscopy (n = 3; 6.3%); hemoglobin A1C measurement, fetal Doptone, streptococcus diagnostic testing, and typhoid diagnostic testing (each n = 2; 4.2%); erythrocyte sedimentation rate (ESR) tests, fluorescein strips, gram stain, Hepatitis B and C testing, fecal occult blood tests, serum protein, sickle cell testing, tuberculosis testing, and thyroid stimulating hormone (each n = 1; 2.1%).

### Associations between NGO characteristics and POC tests

Secular groups were more likely to carry portable ultrasound (p = 0.028), but there was no significant association between group motivation (faith-based versus secular) and likelihood of carrying any of the other POC tests (glucometer, p = 0.691; urine dipsticks, p = 0.462; qualitative urine pregnancy tests, p = 0.516; hemoglobinometer, p = 0.516; malaria testing, p = 0.533; STI testing, p = 1.000). There was no significant association between the number of MSTs dispatched by the organization each year (more than three versus three or fewer) and the likelihood of carrying any POC diagnostic test (glucometer, p = 1.000; urine dipsticks, p = 0.484; qualitative urine pregnancy tests, p = 1.000; hemoglobinometer, p = 0.744; malaria testing, p = 0.208; STI testing, p = 1.000; portable ultrasound, p = 0.538).

## Discussion

This is the first study to describe the availability of selected POC tests on mobile clinics offered by MST-sending organizations. Our results indicate that glucometers, urine dipsticks, and qualitative urine pregnancy tests are commonly available, and their ubiquity may make availability a basic expectation among prospective volunteer clinicians. Despite concerns that faith-based groups may be underrepresented in the academic literature due to competing organizational motivations [1] we found no evidence that this underrepresentation equates to diminished preparation with POC diagnostic tests, nor did we find evidence that groups that operate mobile clinics less frequently arrive less prepared.

POC tests used on such brigades should ideally be targeted to common and important (i.e. those associated with long-term morbidity and mortality) medical conditions and appropriate clinical guidelines for MSTs should likewise be grounded in an understanding of what tests are reasonably available to most brigades. With broad similarities in epidemiology across many mobile MST locations in LAC [9,10], the above specified POC tests would be useful in 7.9–24.6% of complaints (diabetes, pregnancy, urinary tract infection, dyspepsia) assessed by clinicians. Given this potential to either benefit or harm up to one in four MST patients, clinicians should be knowledgeable about the performance characteristics of the tests that they are using.

Knowledge of local epidemiology is particularly important for estimating the necessary resources for diagnosis and treatment in mobile clinics, as well as for the development of appropriate diagnostic protocols. Theoretical concerns for MST-sending organizations include the use of POC tests by unqualified personnel, and the implications of relying on the accuracy of such tests in remote locations with potentially limited clinical follow-up. Furthermore, organizations must consider the accuracy of their own test brands, the cost of such tests, and whether they should be rationed to the patients who would benefit most. For example, a policy of universal glucometer and hemoglobinometer screening for diabetes and anemia respectively, or malaria and chlamydia tests for all those with subjective fever or vaginal discharge would strain the resources of sending organizations, and potentially commit asymptomatic patients to treatment and follow-up that lies beyond their means.

The following sections provide an overview of important performance characteristics of the most commonly carried POC tests on mobile MSTs, which are further summarized in Table 2.

**Table 2:** Common POC tests used on mobile MSTs in Latin America and the Caribbean, the associated disease entities they are used to diagnose, the approximate prevalence of that disease entity on MSTs [9,10], the sensitivity and specificity of the test, and the approximate cost per test (obtained from allmedtech.com on December 10, 2016).

Point of care test	Diagnostic use on MSTs	Prevalence of diagnosis on MSTs in LAC	Accuracy	Approximate cost

**Glucometer**	Diabetes	0.3–2.3%	Similar to gold standard laboratory testing	$1.19/test ($59.99/50 tests); Accucheck Aviva Plus glucose test strips
**Urine dipsticks**	Urinary tract infection	1.8–2.6%	Leukocyte esterase sensitivity 14–100%, specificity 25–86%; nitrites: sensitivity 14–80%, specificity 58–100%	$0.45/test ($45/100 reagent strips); Siemens Multistix
**Qualitative urine pregnancy**	Pregnancy	0.4–1.9%	Sensitivity 53–78% for BHCG 20–300 (approximately 4 weeks gestation)	$0.34/test ($34.99/100 tests; New Choice Pro)
**Hemoglobinometer**	Anemia	0.5–1.3%	HemoCue 95% confidence range: 1.52–2.7g/dL below actual Hb, to 1.79–2.7g/dL above actual Hb	$1.67 ($335/200 tests); HemoCue microcuvettes for HB201 Plus analyser)
**Chlamydia test**	Vaginal discharge	1.1–3.6%	Sensitivity 67–74%; specificity 91–96%	$11/test ($220/20 tests); Clearview chlamydia test
**Portable ultrasound**	Pregnancy, abdominal pain	0.4–1.9%; 7.4–11%	User dependent	$8250 base price; Sonimage P3 handheld ultrasound

### Urine dipsticks

Despite their ubiquitous use by respondents to this survey, urine dipsticks have a wide accuracy range when compared with the gold standard of a urine culture to diagnose UTI. In previous studies, the sensitivity and specificity have varied markedly based on whether leukocyte esterase, nitrites, or both are used as clinical diagnostic criteria [11,12,13,14,15,16,17,18,19,20,21]. Clinicians should be aware of the accuracy limitations of relying solely on urine dipstick results for treatment decisions, and accordingly might consider placing greater emphasis on clinical gestalt. While there is scant published evidence of the use of clinical guidelines on MSTs [5], one study does nonetheless suggest a combination of clinical criteria (dysuria, frequency, urgency) and a positive urine dipstick leukocyte esterase as sufficient for diagnosis in an MST context [22].

### Qualitative urine pregnancy tests

Qualitative urine pregnancy tests have variable accuracy, with frequent false negatives in early pregnancy and in specimens with low specific gravity. Most tests claim to detect BhCG at a level of 25 mIU/ml, but in a Belgian trial, only three out of eight available tests achieved this performance. A positive test should be present three to four days following implantation, with 98% positive at seven days [23]. For a BhCG of 20–300 (approximately four weeks pregnant), the OSOM and QuickVue brand tests detected early pregnancy 53% and 78% of the time, respectively [24]. The sensitivity of home pregnancy tests, likewise, varied between 75–91% in one study [25], depending on whether samples were tested by volunteers or by patients themselves. Therefore, MST clinicians should consider the uncertain performance of such tests in the context of a convincing patient history of missed menses, or potentially serious symptoms such as acute pelvic pain or vaginal bleeding, which could indicate serious pathology such as ectopic pregnancy.

### Glucometers

POC glucometers appear to be ubiquitous on MSTs, and their use for diagnosis is well supported by major international guidelines [26,27], with the diagnosis suggested by a fasting glucose >7 mmol/dL, >11.1 mmol/dL two-hour glucose tolerance, or random blood glucose >11.1 mmol/dL with symptoms [26,27]. A borderline result should be repeated, particularly if the patient is asymptomatic [26]. The same guidelines suggest that in resource limited settings, detection programs should be opportunistic and limited to high risk individuals [26], a strategy that would avoid placing further strain on the financial and logistic resources of MSTs.

### Hemoglobinometers

Although specific brands used were not collected in this study, hemoglobinometers are a comparatively expensive diagnostic tool, and were less commonly used by MST-sending organizations than other POC tests. Furthermore, their accuracy may be questionable, given comparisons of POC hemoglobinometers with gold standard laboratory readings, that indicated a difference greater than 1 g/dl in 21–30.8% of measurements in the case of the Hemocue [28,29,30]. The Hemocue consistently overestimated hemoglobin by amounts that may be clinically significant [28]. This would suggest a low threshold for MSTs to initiate iron, folic acid, or antiparasitic therapy in patients with clinically suspected anemia.

The World Health Organization (WHO) hemoglobin color scale uses absorbent chromatography paper and matches the color of a drop of blood against a standardized scale. In a study of 548 outpatients in Johannesburg, sensitivity and specificity of this tool were 96% and 86% respectively [31] and 91% and 86% in another [32], while the cost per test was one tenth that of photometric analysis [32]. An additional, although less accurate noninvasive colorimetric instrument contrasts the color of the conjunctiva against that of the instrument, and had a sensitivity and specificity of 63% and 72% respectively [33]. Such inexpensive, portable tools might be valuable to organizations that cannot afford more expensive hemoglobinometers.

### Limitations

Weaknesses of this study include the use of an unvalidated survey tool to collect data and that data collection may have been limited by the knowledge of the person answering the survey, who may not have been a clinician. This might have the effect of underestimating the POC tests carried by MSTs. The findings may not be generalizable to other types of MSTs (e.g. established clinics, specialized services, surgical missions, military missions). We were unable to compare the availability with the actual rates of use of these POC tests within the medical brigades.

### Conclusions and future directions

Most mobile MSTs serving Latin America carry urine dipsticks, qualitative urine pregnancy tests, and glucometers. MST-sending organizations and volunteer clinicians share responsibility for understanding the performance characteristics of the tests that they carry, and this should be a focus in pre-brigade curriculum. Future development of guidelines for clinical care on MSTs should be based on an awareness of the diagnostic resources that are at the disposal of most volunteer groups.
